# Monitoring agroforest plots under the scope of the COMCHA project. 1. Baseline data for the vascular plants and arthropods of "Vale da Fonte Plot" in Pico Island (Azores Archipelago)

**DOI:** 10.3897/BDJ.14.e174244

**Published:** 2026-02-18

**Authors:** Alexandra Dal Lago, Andrea Petrone, António O. Soares, Mário Boieiro, David H. Lopes, Tiago Freitas, Sébastien Lhoumeau, Sophie Wallon, Paulo A. V. Borges

**Affiliations:** 1 University of Bologna, Bologna, Italy University of Bologna Bologna Italy; 2 University of Azores, CE3C—Centre for Ecology, Evolution and Environmental Changes, Azorean Biodiversity Group, CHANGE —Global Change and Sustainability Institute, Faculty of Science and Technology, Rua da Mãe de Deus, 9500-321, Ponta Delgada, São Miguel, Azores, Portugal University of Azores, CE3C—Centre for Ecology, Evolution and Environmental Changes, Azorean Biodiversity Group, CHANGE —Global Change and Sustainability Institute, Faculty of Science and Technology, Rua da Mãe de Deus, 9500-321 Ponta Delgada, São Miguel, Azores Portugal; 3 IUCN SSC Atlantic Islands Invertebrate Specialist Group, Angra do Heroísmo, Azores, Portugal IUCN SSC Atlantic Islands Invertebrate Specialist Group Angra do Heroísmo, Azores Portugal; 4 University of Azores, CE3C—Centre for Ecology, Evolution and Environmental Changes, Azorean Biodiversity Group, CHANGE —Global Change and Sustainability Institute, School of Agricultural and Environmental Sciences, Rua Capitão João d’Ávila, Pico da Urze, 9700-042, Angra do Heroísmo, Azores, Portugal University of Azores, CE3C—Centre for Ecology, Evolution and Environmental Changes, Azorean Biodiversity Group, CHANGE —Global Change and Sustainability Institute, School of Agricultural and Environmental Sciences, Rua Capitão João d’Ávila, Pico da Urze, 9700-042 Angra do Heroísmo, Azores Portugal; 5 LIBRe – Laboratory for Integrative Biodiversity Research, Finnish Museum of Natural History, University of Helsinki, Helsinki, Finland LIBRe – Laboratory for Integrative Biodiversity Research, Finnish Museum of Natural History, University of Helsinki Helsinki Finland; 6 Vale da Fonte, São Roque, Azores, Portugal Vale da Fonte São Roque, Azores Portugal; 7 IUCN SSC Monitoring Specialist Group, Angra do Heroísmo, Azores, Portugal IUCN SSC Monitoring Specialist Group Angra do Heroísmo, Azores Portugal

**Keywords:** Azores, coastal native forest mosaic, COMCHA project, island biodiversity, Pico Island, plot-based monitoring, pollinators, terrestrial arthropods, vascular plants.

## Abstract

**Background:**

Agroforestry is increasingly promoted as a nature-based solution (NbS) capable of reconciling production with biodiversity conservation, particularly in island landscapes, where endemism and fragmentation heighten conservation stakes. We established a permanent agroforest monitoring plot at Vale da Fonte (Pico Island, Azores) to generate baseline data on vascular plants and terrestrial arthropods and to evaluate whether a structurally complex orchard embedded in native coastal forest can support native and endemic biotas and key ecosystem functions. We used a modified COBRA framework integrating complementary active protocols (nocturnal aerial searching, day/night beating and day sweeping), together with pollinators time transects, pan traps and targeted pest traps. We collected 69 standardised samples (5 plant, 64 arthropod) and made the dataset publicly available in Darwin Core format via the GBIF IPT for long-term use.

**New information:**

The botanical survey documented 155 vascular plant species including 12 taxa recorded for the first time for Pico Island. These newly-recorded species are: Centaurium tenuiflorum (Hoffm. & Link) Fritsch subsp. tenuiflorum (Naturalized), *Lolium rigidum* Gaudin (Naturalised), *Malus domestica* Borkh (Casual), *Mentha spicata* L. (Naturalised), *Monstera deliciosa* Liebm. (Casual), *Opuntia ficus-indica* (L.) Mill. (Naturalised), *Passiflora edulis* Sims (Casual), *Poa pratensis* L. (Casual), *Prunus armeniaca* L. (Casual), *Scrophularia auriculata* L. (Casual), *Trifolium pratense* L. (Naturalised) and *Verbena rigida* Spreng. (Naturalised).

We recorded 169 arthropod taxa from 18 orders and 84 families, comprising 150 identified taxa (56 native non-endemic, 14 endemic, 74 introduced and six of indeterminate status). We also documented a total of 18 first records to Pico Island, one of which is also a new record for the Azores Archipelago, the European paper wasp Polistes dominula (Christ, 1791) (Hymenoptera, Vespidae).

In addition, these results highlight the mixed native–exotic character of lowland agroforestry mosaics on oceanic islands and provide a baseline for future monitoring of biosecurity risks, restoration efficacy and ecosystem-service provisioning in insular production landscapes.

## Introduction

Biodiversity is in a global crisis: rapid losses of species, ecosystem functions and evolutionary potential are undermining resilience and the services people depend on, with over 47,000 species now threatened with extinction (Mace et al. 2012, Borges et al. 2019, IUCN 2025). These declines are largely driven by human pressures, namely land-use change and fragmentation, overexploitation, invasive species, pollution and climate change and are often subtle and long-term, making them hard to perceive and prioritise politically (Pascual et al. 2021).

Agroforestry is defined as the intentional integration of trees with crops (or livestock) in multifunctional systems that deliver multiple ecosystem services (Garrity 2004). It shapes an agroecosystem that can simultaneously support high agricultural productivity and biodiversity goals (Garrity 2004). By enhancing intra- and interspecific diversity amongst trees, agroforestry systems foster ecological resilience, stabilise income through year-round provisioning of goods and reduce seasonal fluctuations in productivity (Quandt et al. 2019). Agroforestry is, thus, both an ecological and social strategy, fully aligned with the principles of agroecology — an integrated, territorial and bottom-up approach to transforming food systems through the co-creation of knowledge and the empowerment of producers (Tscharntke et al. 2011). Shaded perennial agroforestry systems, such as those involving *Coffea arabica* L. and *Theobroma cacao* L., regulate microclimate, improve soil and water efficiency and enhance nutrient cycling. By increasing structural and species diversity, they promote ecological resilience (Gomes et al. 2020). Examples from Brazil and Rwanda show that such systems can maintain crop yields, support biodiversity and deliver social and climate benefits, making agroforestry a scalable and multifunctional nature-based solutions (NbS) (Souza et al. 2012, Byiringiro et al. 2024).

Insular systems, such as the Azores Archipelago, provide a compelling case study of how anthropogenically altered habitats, including pastures, orchards and exotic forests, can influence native biodiversity in complex and sometimes contradictory ways (Cardoso et al. 2009). Due to their usually high levels of endemism and ecological sensitivity, island ecosystems like those of the Azores Archipelago are particularly vulnerable to landscape transformation (Oyarzabal et al. 2025). One example of such mosaic systems is represented by lowland exotic forest patches embedded within or adjacent to native forest remnants (Tsafack et al. 2021). These mosaics form transitional zones where native and anthropogenic elements interact, potentially supporting refuges or dispersal corridors for endemic species across fragmented landscapes (Cardoso et al. 2009, Marcelino et al. 2021, Tsafack et al. 2021). In this context, the layered vegetation, deadwood and microhabitat heterogeneity of these semi-natural stands create a permeable matrix that enables native species to move amongst remnants, thereby maintaining functional landscape connectivity (Aparício et al. 2018). At the same time, however, these same habitats often host introduced plant species and are exposed to edge effects, making them vulnerable to biological invasions that threaten the persistence of native taxa (Borges et al. 2022a). Similarly, small lowland patches of exotic forests, such as *Cryptomeria japonica* (L.f.) D.Don plantations and *Pittosporum undulatum* Vent. forest patches, can temporarily serve as refuges for rare endemic arthropods (Tsafack et al. 2021, Lhoumeau et al. 2025). Although these human-made habitats cannot substitute the ecological value of native montane cloud forests, they can contribute to short-term persistence and functional connectivity between fragmented native patches (Cardoso et al. 2009, Tsafack et al. 2021, Borges et al. 2022a). Taken together, this reinforces the idea that mosaic landscapes composed of native remnants interspersed with anthropogenic habitats are ecologically significant, albeit imperfect, components of modern insular ecosystems.

The Vale da Fonte, located on Pico Island (Azores Archipelago), is a recent innovative project aimed at establishing of an agroforestry system. This project aims to create the intentional integration of trees and shrubs (including native plants from the Azores Archipelago) into crop and animal farming systems to create environmental, economic and social benefits. In these systems, it is expected that the combination of agriculture and forestry creates more integrated, diverse, productive, profitable, healthy and sustainable land-use systems. Research conducted in Europe shows important benefits from agroforestry to the protection of biodiversity, enhancing ecosystem services and preserving habitats and landscapes by providing food, shelter and habitat for birds and insects; moreover, pesticide applications that harm insect populations are reduced or abolished (Augère-Granier 2020).

## General description

### Purpose

This dataset provides an inventory of auxiliary insects and pest species in a structurally complex orchard embedded within a native coastal forest matrix on Pico Island (Azores). The study aims to assess whether such intermingling agroecosystems can support native and endemic arthropod biodiversity, functioning as refuges or dispersal corridors. As part of a long-term monitoring project, permanent plots were established to collect standardised data on plant and arthropod assemblages. The resulting dataset contributes to evaluating semi-natural orchards as potential Nature-based Solutions (NbS) and provides a baseline for biodiversity assessments in anthropogenically influenced landscapes across the Azores Archipelago.

## Project description

### Title

Monitoring agroforest plots under the scope of the COMCHA project in the Vale da Fonte Plot in Pico Island (Azores)

### Personnel

The project was conceived by Paulo A.V. Borges.

Fieldwork: (Pico Island) - Alexandra Dal Lago, Andrea Petrone, António Onofre Soares, Mário Boieiro, Paulo A. V. Borges, Tiago Freitas.

Fieldwork permits: As the study site lies outside the Pico Island Natural Park, no permit was necessary. Sampling was performed with the landowner’s authorisation (Tiago Freitas, co-author; BIODIVERSA+ COMCHA partner).

Parataxonomists: Alexandra Dal Lago, Sophie Wallon.

Taxonomists: Andrea Petrone (Vascular Plants), António Onofre Soares (Hemiptera Pests), David João Horta Lopes (Thrips), Luís Carlos Crespo (Confirmation of some Araneae); Mário Boieiro (Pollinators), Paulo A. V. Borges (COBRA arthropod sampled species), Sophie Wallon (Pests).

Database management: Andrea Petrone, Paulo A. V. Borges and Sébastien Lhoumeau.

Darwin Core Database management: Andrea Petrone, Mário Boieiro, Paulo A. V. Borges and Sébastien Lhoumeau.

### Study area description

**Figure 1.**
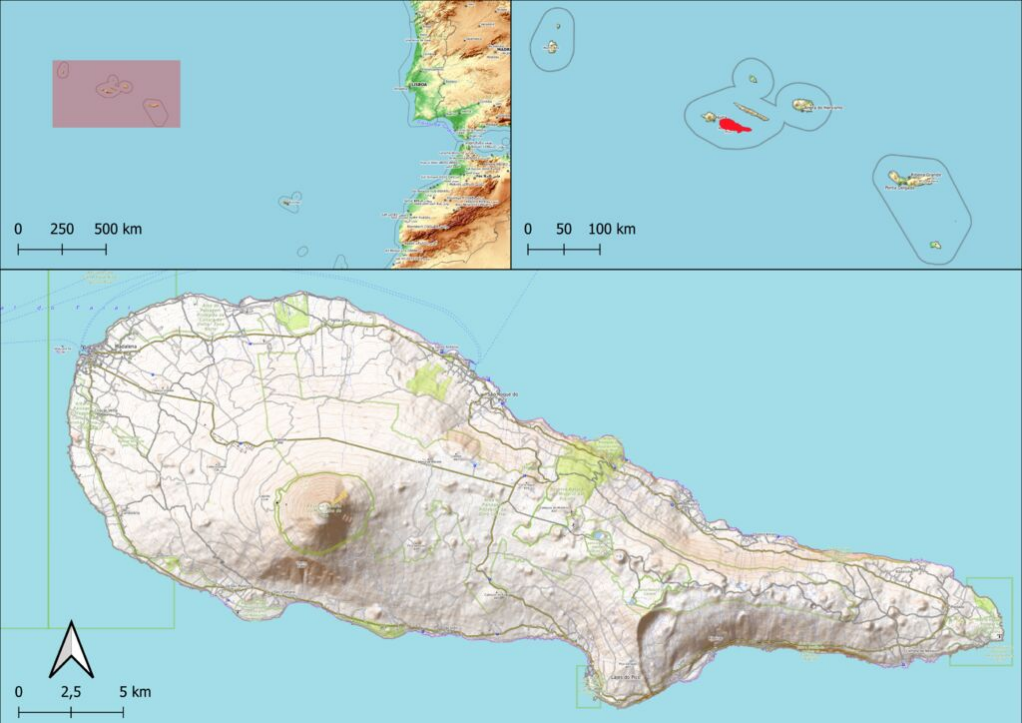
Location of the Azores Archipelago in the mid-Atlantic (top left), the position of Pico Island within the Archipelago marked in red (top right) and a detailed view of Pico Island (bottom) (credit: Alexandra Dal Lago).

Pico Island is located in the central group of the Azores Archipelago (Portugal), in the North Atlantic Ocean, between 38°33′57″ and 38°33′44″ N and 28°01′39″ and 28°32′33″ W (Fig. 1). It is the second-largest island of the Archipelago, with an area of approximately 445 km² and a coastline of 151.8 km in length and represents the highest point in Portugal with Mount Pico reaching 2,351 m a.s.l. (Gil et al. 2017, Coelho et al. 2021). Geologically, it is also the youngest island in the Archipelago, with an estimated age between 0.19 and 0.27 million years (Coelho et al. 2021, Borges et al. 2021). Pico has a temperate oceanic climate with mild winters and rare frost at low elevations, abundant year-round rainfall and high relative humidity that increases with altitude; winds are persistent, especially in autumn and winter (Gil et al. 2017, Coelho et al. 2021). The landscape of the Island forms a concentric pattern shaped by both natural and anthropogenic land uses along an altitudinal gradient. Lowland coastal areas are primarily occupied by urban settlements and intensive agriculture, while mid-elevations are largely dominated by pastures. In contrast, the central highlands are characterised by more extensive and preserved natural habitats. Over time, the original vegetation, once dominated by native forests (including the dominant Laurel forest at mid-elevation), has been significantly altered, with large areas being converted into exotic plantations, croplands and urban zones. Particularly during the late 20^th^ century, widespread deforestation occurred at mid- and high elevations to make way for pastureland, contributing to the ongoing fragmentation of native ecosystems (Gil et al. 2017, Borges Silva et al. 2023). Pico has the largest modelled extent of potential coastal woodland in the Archipelago; submontane laurel forests (Laurus) could occupy an estimated 49–65% of the Island. *Juniperus*-*Ilex* montane forests could potentially cover 20% of its surface. Unique within this Archipelago, subalpine scrublands dominated by *Calluna vulgaris* and *Erica azorica* occur only on Pico, between 1200 and 1700 m a.s.l., representing vegetation types that have experienced minimal anthropogenic disturbance (Elias et al. 2016).

### Design description

A modified version of the COBRA protocol (Borges et al. 2018) was applied to guide arthropod sampling. This protocol was adapted to combine multiple standardised techniques aimed at maximising arthropod detection across various microhabitats and temporal windows. The sampling effort included a combination of nocturnal aerial sampling, diurnal sweeping and both day and night beating. To specifically target pollinating insects, soil-level and elevated pan traps were used in combination with standardised transect sampling. Vegetation composition within the orchard was assessed using a random-walk (meander) botanical survey that systematically traversed the area to maximise detectability, covering all five plots over five field days.

A total of 69 samples were collected (five for plants and 64 for arthropods). All arthropod specimens were subsequently sorted and identified by trained parataxonomists and taxonomists. The dataset was standardised according to Darwin Core standards to promote consistency, interoperability and long-term usability. The study area was further divided into five plots, each characterised by distinct vegetation features.

### Funding

Fieldwork was financed by the project EU BIODIVERSA + COMCHA Community-based change: local and traditional knowledge(s) in NbS, through a Grant from Fundo Regional da Ciência e Tecnologia (FRCT). Open data availability for the general public will be available through funding from AZORES BIOPORTAL, funded by FEDER at 85% and by regional funds at 15%, via the Azores 2020 Operational Programme, through the “PORBIOTA-AZORES BIOPORTAL” project (ACORES-01-0145-FEDER-000072) (2019-2022) and also the project “Azores Bioportal – Biodiversity Portal of the Azores” (FRCT M1.1.A/INFRAEST CIENT/001/2022) (2022-2023).

## Sampling methods

### Study extent

**Figure 2.**
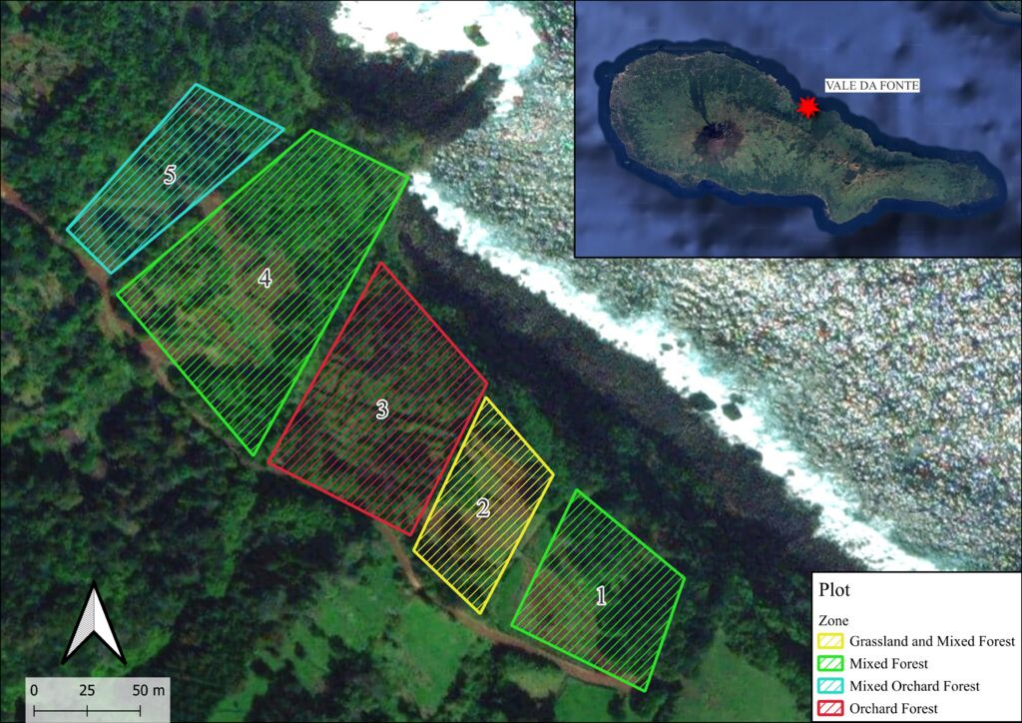
Location of the study area - Vale da Fonte - on Pico Island (top right) and distribution of the five sampling plots, based on Google Earth. Numbers from 1 to 5 indicate the sampling plots, with colours denoting different habitat types, namely Mixed Forest (1 and 4), Grassland and Mixed Forest (2), Orchard Forest (3) and Mixed Orchard Forest (5) (credit: Alexandra Dal Lago).

This study was carried out in Vale da Fonte, located on Pico Island (Azores Archipelago), within a structurally complex orchard embedded in a surrounding matrix of native coastal forest. Fieldwork took place mostly between 16 and 19 June 2025, across five distinct plots, situated at 38.5027707 N, -28.27836457 W and spanning an elevation range of 72 to 83 m a.s.l. The selected zones encompassed a range of habitat types, including areas of mixed native and exotic forest (Zone 1 and Zone 4), open grassland interspersed with patches of mixed forest (Zone 2), orchard-dominated forest with scattered fruit trees and native vegetation (Zone 3) and a transitional zone between orchard and forest, characterised by a combination of cultivated and spontaneous plant species (Zone 5) (Fig. 2).

### Sampling description

In this study, optimised and standardised sampling protocols were applied to assess terrestrial arthropod diversity within a structurally complex orchard on Pico Island. The fieldwork was conducted during the summer season, a period corresponding to the peak of vegetation and insect development. For arthropods, a total of 64 samples were collected across five plots using a combination of active and passive sampling methods, namely:

- Active Aerial Searching (AAS) was implemented during the nocturnal period to collect arthropods above knee-level. This method involved manually capturing visible arthropods using forceps, pooters or brushes. Each session lasted one hour per researcher. The AAS method targeted a wide range of functional groups, including predatory arthropods (e.g. spiders, true bugs, beetles), phytophagous insects and saprophagous taxa, such as millipedes and detritivorous beetles. In total, 10 samples were collected using this method.

- Foliage Beating (FB) was conducted to dislodge arthropods from the canopy and understorey vegetation. During daylight hours, plots were sampled using a standard 110 cm × 80 cm framed beating sheet. A wooden pole (≥ 1.5 m) was used to vigorously strike the branches to dislodge arthropods, which fell on to the cloth for collection. The same technique was applied during nocturnal periods, for a total of 10 night-beating and 5 day-beating samples.

- Foliage Sweeping (FS) targeted herbaceous vegetation and shrubs during daytime using a sweep net with a 46 cm diameter opening. A total of five sweeping samples were collected, each lasting one hour.

- To monitor the medfruitfly *Ceratitis capitata* (Wiedemann, 1824) (Diptera, Tephritidae), we used the tephri trap with the commercial food attractant Trimedlure. The lure is placed inside a green cage with the black top facing upwards, ensuring a gradual release of the attractant over several weeks. Traps were typically deployed at approximately 1 m above ground level, positioned near host vegetation to maximise capture efficiency. Traps were deployed at standardised heights — one in plot 3 and two in plot 5 — and left in the field for around 40 days to maximise capture efficiency and ensure representative sampling of the local frugivorous insect community.

- Thrips Survey was conducted using yellow sticky traps placed at standardised positions on host plants, in the plots 3 and 5 for 40 days. These sticky traps exploit the attraction of thrips to bright yellow surfaces, capturing individuals passively as they land.

- The banana weevil *Cosmopolites sordidus* (Germar, 1824) (Coleoptera, Curculionidae) was monitored using CosmoTrack pheromone pitfall traps. Each trap is a yellow cylindrical device (radius: 15 cm; height: 14 cm) composed of two compartments separated by a central opening of 1.5 cm. The lower half is buried in the soil, while the upper half remains above ground, allowing adult weevils to enter through the central opening. Traps were baited with the aggregation pheromone *Cosmoplus*, placed inside the trap, to attract adults. Once inside, individuals were unable to escape. Pitfalls were deployed, one in plot 3 and one in plot 5, for 47 days to maximise the capture efficiency and provide reliable estimates of adult *C. sordidus* activity.

To monitor pollinators, two complementary methods were employed: pan trapping and timed-transect sampling. Pan trapping consisted of placing two sets of pan traps per plot, distanced by ~ 50 m. One set was placed at soil level, while in the other, the traps were levelled at the average flower height. Each set comprised three plastic bowls (11 cm diameter) painted blue, yellow and white to attract different pollinator taxa. The traps were filled with water and detergent to break surface tension and remained in the field for two full days before specimen retrieval. In total, five vertical and four soil-level pan-trap sets were deployed.

Timed-transect sampling involved walking randomly throughout each plot under optimal weather conditions for pollinator activity (i.e. sunny or partly cloudy, without wind or precipitation) allowing a comprehensive spatial coverage of the area. During each transect, pollinator observations and captures using a sweep net were conducted during a 30-minute period. Two replicate transects were carried out in each plot; thus a total of ten transect samples were obtained.

All collected specimens were preserved in 96% ethanol and later processed in the laboratory. Arthropods were sorted to morphospecies and identified by trained parataxonomists and expert taxonomists.

In parallel with arthropod sampling, a comprehensive botanical survey was conducted by one of us (Andrea Petrone) to characterise the vegetation composition within each plot. A field operator performed in situ plant identification using regional floras and taxonomic keys. This ensured complete documentation of the plant community associated with each arthropod sampling unit.

Collected data were standardised according to Darwin Core biodiversity data standards and quality control measures included taxonomic verification and standardised georeferencing.

### Quality control

Arthropod specimens collected using pan traps and sweeping nets were transported to the laboratory, where individuals were sorted and identified to species level using a stereomicroscope and specific taxonomic literature. Pollinator specimens were identified by Mário Boieiro, while the remaining arthropods were initially sorted by parataxonomist Alexandra Dal Lago and subsequently identified by Paulo A. V. Borges. Juvenile individuals were included in the dataset, as the low species diversity in the Azores allows for reliable identification. Pest species were identified by António O. Soares.

Plant species were assigned taxonomic ranks and conservation statuses, based on information available from the Azores Bioportal – PORBIOTA, the official biodiversity platform for the Azores Archipelago, which provides validated and up-to-date data on the region’s flora, including endemism, invasiveness and habitat preference.

### Step description

A reference collection of Azorean arthropods, housed at the Dalberto Teixeira Pombo Insect Collection (University of the Azores), was used to help specimen identification. The taxonomic nomenclature and colonisation status of the species follows the most recent checklist of Azorean arthropods (Borges et al. 2022b).

## Geographic coverage

### Description

Pico Island, Azores Archipelago, Portugal.

### Coordinates

38.502 and 38.504 Latitude; -28.279 and -28.277 Longitude.

## Taxonomic coverage

### Description

The following phyla, classes (in bold) and families of plants are covered:

Phylum Lycopodiophyta

**Selaginellopsida**: Selaginellaceae

Phylum Pteridophyta

**Polypodiopsida**: Athyriaceae, Blechnaceae, Cyatheaceae, Dennstaedtiaceae, Dryopteridaceae, Polypodiaceae, Pteridaceae, Thelypteridaceae.

Phylum Pinophyta

**Pinopsida**: Araucariaceae, Cupressaceae.

Phylum Magnoliophyta

**Liliopsida**: Araceae, Arecaceae, Asparagaceae, Bromeliaceae, Cannaceae, Commelinaceae, Cyperaceae, Iridaceae, Juncaceae, Musaceae, Poaceae, Smilacaceae, Strelitziaceae, Zingiberaceae.

**Magnoliopsida**: Anacardiaceae, Annonaceae, Apiaceae, Apocynaceae, Araliaceae, Asteraceae, Brassicaceae, Cactaceae, Caryophyllaceae, Convolvulaceae, Crassulaceae, Ebenaceae, Ericaceae, Euphorbiaceae, Fabaceae, Gentianaceae, Geraniaceae, Hydrangeaceae, Hypericaceae, Juglandaceae, Lamiaceae, Lauraceae, Lythraceae, Malvaceae, Moraceae, Myricaceae, Myrsinaceae, Myrtaceae, Ochnaceae, Oleaceae, Onagraceae, Orobanchaceae, Oxalidaceae, Passifloraceae, Phytolaccaceae, Pittosporaceae, Plantaginaceae, Polygonaceae, Primulaceae, Proteaceae, Rhamnaceae, Rosaceae, Rubiaceae, Rutaceae, Salicaceae, Scrophulariaceae, Solanaceae, Verbenaceae, Vitaceae.

The following classes (in bold) and orders of arthropods are covered:

**Arachnida**: Araneae, Opilliones, Pseudoscorpiones;

**Chilopoda**: Lithobiomorpha, Scutigeromorpha;

**Diplopoda**: Julida;

**Insecta**: Archaeognatha, Coleoptera, Dermaptera, Diptera, Hemiptera, Hymenoptera, Lepidoptera, Odonata, Orthoptera, Phasmida, Psocodea, Thysanoptera.

## Temporal coverage

**Data range:** 2025-5-23 – 2025-8-18.

### Notes

The principal survey of arthropods and vascular plants occurred during 16-06-2025 to 20-06-2025; traps for fruit flies, thrips and the banana weevil remained active thereafter.

## Collection data

### Collection name

Entomoteca Dalberto Teixeira Pombo at University of Azores.

### Collection identifier

DTP

### Specimen preservation method

All specimens were preserved in 96% ethanol.

### Curatorial unit

Dalberto Teixeira Pombo insect collection at the University of the Azores (Curator: Paulo A. V. Borges).

## Usage licence

### Usage licence

Creative Commons Public Domain Waiver (CC-Zero)

## Data resources

### Data package title

Inventory of vascular plants and arthropods in Vale da Fonte agroforest plot.

### Resource link

https://doi.org/10.15468/ag5j58

### Alternative identifiers

https://www.gbif.org/dataset/67301bd7-3844-4858-81a4-2f2cab5c42f5

### Number of data sets

2

### Data set 1.

#### Data set name

Event table

#### Data format

Darwin Core Archive format

#### Character set

UTF-8

#### Download URL

https://ipt.gbif.pt/ipt/resource?r=biotavalefonte

#### Data format version

Version 1.1

#### Description

The dataset was published in the Global Biodiversity Information Facility platform, GBIF (Borges et al. 2025). The following data table includes all the records for which a taxonomic identification of the species was possible. The dataset submitted to GBIF is structured as a sample event dataset that has been published as a Darwin Core Archive (DwCA), which is a standardised format for sharing biodiversity data as a set of one or more data tables. The core data file contains 69 records (eventID). This GBIF IPT (Integrated Publishing Toolkit, Version 2.5.6) archives the data and, thus, serves as the data repository. The data and resource metadata are available for download in the Portuguese GBIF Portal IPT.

**Data set 1.** 

Column label	Column description
eventID	Identifier of the events, unique for the dataset.
locationID	Identifier of the location.
habitat	The surveyed habitat.
fieldNumber	An identifier given to the event in the field. Often serves as a link between field notes and the Event.
locationRemarks	Details on the locality site.
samplingProtocol	The sampling protocol used to capture the species.
sampleSizeValue	The numeric amount of time spent in each sampling.
sampleSizeUnit	The unit of the sample size value.
samplingEffort	The amount of time of each sampling.
eventDate	Date or date range the record was collected.
year	Year of the event.
month	Month of the event.
day	Day of the event.
continent	Name of the continent.
islandGroup	Name of archipelago.
island	Name of the island.
country	Country of the sampling site.
municipality	Municipality of the sampling site.
locality	Name of the locality.
locationRemarks	Details on the locality site.
minimumElevationInMetres	The lower limit of the range of elevation (altitude, usually above sea level), in metres.
decimalLatitude	Approximate centre point decimal latitude of the field site in GPS coordinates.
decimalLongitude	Approximate centre point decimal longitude of the field site in GPS coordinates.
geodeticDatum	The ellipsoid, geodetic datum or spatial reference system (SRS), upon which the geographic coordinates given in decimalLatitude and decimalLongitude are based.
coordinateUncertaintyInMetres	Uncertainty of the coordinates of the centre of the sampling plot.
coordinatePrecision	Precision of the coordinates.
georeferenceSources	A list (concatenated and separated) of maps, gazetteers or other resources used to georeference the Location, described specifically enough to allow anyone in the future to use the same resources.
datasetName	Name of the dataset.

### Data set 2.

#### Data set name

Occurrence table

#### Data format

Darwin Core Archive format

#### Character set

UTF-8

#### Download URL

https://ipt.gbif.pt/ipt/resource?r=biotavalefonte

#### Data format version

Version 1.1

#### Description

The dataset was published in the Global Biodiversity Information Facility platform, GBIF (Borges et al. 2025). The following data table includes all the records for which a taxonomic identification of the species was possible. The dataset submitted to GBIF is structured as an occurrence table that has been published as a Darwin Core Archive (DwCA), which is a standardised format for sharing biodiversity data as a set of one or more data tables. The core data file contains 1285 records (occurrenceID). This GBIF IPT (Integrated Publishing Toolkit, Version 2.5.6) archives the data and, thus, serves as the data repository. The data and resource metadata are available for download in the Portuguese GBIF Portal IPT.

**Data set 2.** 

Column label	Column description
eventID	Identifier of the events, unique for the dataset.
licence	Reference to the licence under which the record is published.
InstitutionID	The identity of the institution publishing the data.
institutionCode	The code of the institution publishing the data.
basisOfRecord	The nature of the data record.
dynamicProperties	Information on the species colonisation status and also when available the IUCN Red List status.
occurrenceID	Identifier of the record, coded as a global unique identifier.
recordedBy	Name of the person who performed the sampling of the specimens.
identifiedBy	A list (concatenated and separated) of names of people, groups or organisations who assigned the Taxon to the subject.
organismQuantity	A number or enumeration value for the quantity of organisms.
organismQuantityType	The type of quantification system used for the quantity of organisms.
sex	The sex and quantity of the individuals captured.
lifeStage	The life stage of the organisms captured.
establishmentMeans	The process of establishment of the species in the location, using a controlled vocabulary: 'native', 'introduced', 'endemic', "unknown".
dateIdentified	The date on which the subject was determined as representing the taxon.
kingdom	Kingdom name.
phylum	Phylum name.
class	Class name.
order	Order name.
family	Family name.
genus	Genus name.
specificEpithet	Specific epithet.
infraspecificEpithet	Infrapecific epithet.
scientificNameAuthorship	Name of the author of the lowest taxon rank included in the record.
scientificName	Complete scientific name including author and year.
taxonRank	Lowest taxonomic rank of the record.
identificationRemarks	Information about morphospecies identification (code in Dalberto Teixeira Pombo Arthropod Collection).

## Additional information

**Vascular plants**

Our botanical survey documented 155 plant species within the study area. These plants belong to five classes, 34 orders, 73 families and 138 genera. Amongst them, 75 species are naturalised, 23 are native (including 10 endemics), seven are casual introductions and two are of uncertain status. Twelve species are reported for the first time for Pico Island and are new records. Additionally, 48 species were intentionally planted for agricultural purposes. The newly-recorded taxa are as follows: Centaurium tenuiflorum (Hoffm. & Link) Fritsch subsp. tenuiflorum (Naturalised), *Lolium rigidum* Gaudin (Naturalised), *Malus domestica* Borkh (Casual), *Mentha spicata* L. (Naturalised), *Monstera deliciosa* Liebm. (Casual), *Opuntia ficus-indica* (L.) Mill. (Naturalised), *Passiflora edulis* Sims (Casual), *Poa pratensis* L. (Casual), *Prunus armeniaca* L. (Casual), *Scrophularia auriculata* L. (Casual), *Trifolium pratense* L. (Naturalized) and *Verbena rigida* Spreng. (Naturalised).

**Arthropods**

We recorded 169 arthropod taxa across four classes, 18 orders, 84 families and 146 genera. Of these, 150 taxa (89%) were identified to species or subspecies. The 150 identified taxa comprise 14 endemic (9.3%), 56 native non-endemic (37.3%), 74 introduced (49.3%) and six of indeterminate status (4.0%).

Remarkable is the presence of a total of 14 endemic species, including four species mostly dominant in native forest of the Island, namely three spiders (*Rugathodes acoreensis* Wunderlich, 1992; *Gibbaranea occidentalis* Wunderlich, 1989; *Neon acoreensis* Wunderlich, 2008) and the hemiptera *Pinalitus oromii* J. Ribes, 1992. The remaining 10 endemic species are usually also common in several habitats of the Island.

Based on the checklist of Borges et al. (2022b), a total of 18 species were recorded for the first time to Pico Island, one of which is also a new record to the Azores Archipelago (Table 1). The Pico first records are dominated by horticultural and commodity pathways, notably nursery stock (scale insects, thrips), fresh-produce movement (*D. suzukii*, *C. capitata*) and stored products (*S. oryzae*). Most detections come from synanthropic, low-elevation settings (gardens, greenhouses, storage facilities), consistent with island introduction filters and human-mediated dispersal. Species with immediate management relevance include *D. suzukii*, *C. capitata* and *F. occidentalis* due to documented crop impacts and, for the latter, virus transmission. Targeted surveillance in greenhouses, orchards and storage sites, coupled with nursery pathway hygiene, should be prioritised.

Amongst the native species, *Ectomocoris chiragra* (Fabricius, 1803) (Hemiptera, Reduviidae) is a predatory true bug that feeds on other arthropods, contributing to natural biological control within ecosystems. *Eristalis arbustorum* (Linnaeus, 1758) (Diptera, Syrphidae) is a hoverfly that closely resembles the honeybee and plays a key role as a pollinator in both agricultural and natural habitats. Finally, *Xylota segnis* (Linnaeus, 1758) (Diptera, Syrphidae) is another hoverfly, typically occurring in woodlands. Adults feed on nectar and pollen, while larvae develop in decaying plant material, thus contributing to nutrient cycling in forest ecosystems.

The newly -recorded pollinator species are:

*Rivellia syngenesiae* (Fabricius, 1781) - Diptera, Platystomatidae.

This is the first record of the signal fly *Rivellia syngenesiae* in Pico Island. This species was recently recorded in the Azores, after being detected in two localities in Terceira Island (Boieiro et al. 2023). *Rivellia* flies have characteristic wing patterns, but can be confounded with other tephritoid flies, being important to consider both the diagnostic characteristics of the family and more detailed morphological characteristics (Lyneborg 1969, Korneyev 2001, Oosterbroek 2006). *Rivellia syngenesiae* has tarsi predominantly black, the wing band over the posterior cross vein (m-m) is widely separated from the apical spot and the male genitalia are characteristic (Lyneborg 1969). This species is native to Europe, occurring in various countries and their larvae feed on the root nodules of nitrogen-fixing plants (Boieiro et al. 2023), being crucial to assess their potential impact on Azorean native plants.

*Crossocerus elongatulus* (Vander Linden, 1829) - Hymenoptera, Crabronidae.

This is the first record of the slender digger wasp *Crossocerus elongatulus* in Pico Island. This species is known to occur in two other Azorean Islands (Faial and São Miguel), where it has seldom been recorded (Borges et al. 2022b). These digger wasps with a 5-7.5 mm black body, subquadrangular head with bidentate mandibles and the male tergite VII is wide, transverse and with marked punctuation (Bitsch and Leclercq 1993). *Crossocerus elongatulus* has a wide distribution in the Western Palaearctic, occurring in North America, Europe and North Africa. These digger wasps nest in earthy substrates, especially in crevices and prey upon a variety of dipterans, being occasionally found on flowers (Bitsch and Leclercq 1993).

*Polistes dominula* (Christ, 1791) - Hymenoptera, Vespidae.

**Figure 3.**
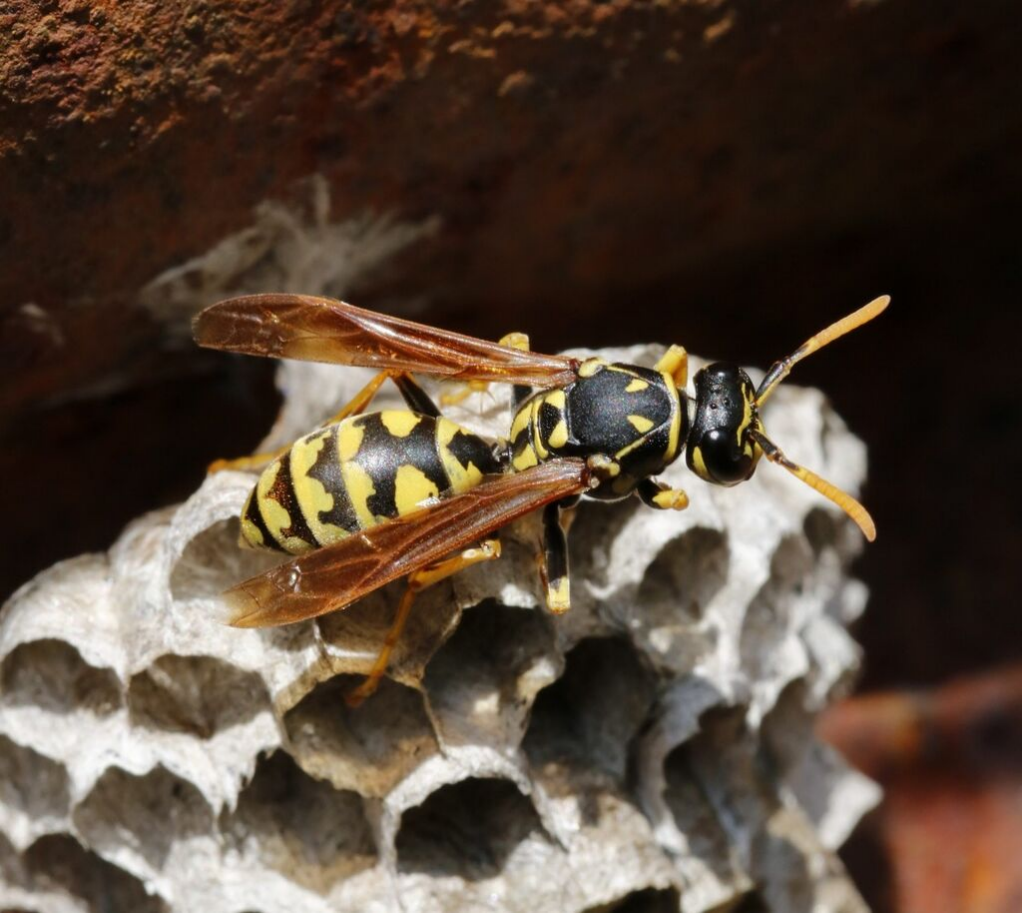
*Polistes dominula* (Christ, 1791) (credit: Alexandra Dal Lago).

This is the first record of the European paper wasp *Polistes dominula* in the Azores (Fig. 3). In Macaronesia, this species is known to occur in the Archipelagos of Madeira and the Canaries, where it is considered a native species (Borges et al. 2008, Arechavaleta et al. 2010). *Polistes dominula* is native to southern Europe, North Africa and western Asia, but the species has been introduced in many territories (North and South America, South Africa, Australia, New Zealand), being considered an invasive species in several countries (Howse et al. (2020) and references therein). These paper wasps (size 12-18 mm) have aposematic colouration and can be recognised by their yellow genae and clypeus (occasionally with black spots or a band), black mandibles (sometimes with a yellow spot), antenna dorsally infuscated up to the 3^rd^ segment then completly orange and apical half of last sternite (VI) yellow (Schmid-Egger et al. 2017, Neumeyer 2019). *Polistes dominula* is a social species, living in nests built with paper and saliva and has a generalist diet. The finding of this species in the Azores is a matter of great concern due to the potential negative consequences for native biodiversity, ecological processes, fruit production and human well-being.Altogether, these results are remarkable: a single small, low-elevation agroforestry plot on Pico yielded 169 arthropod taxa, about 18% of the entire fauna currently known for Pico (928 taxa before this study (Borges et al. 2022b); 946 after adding the 18 new island records reported here) and ~ 7% of all arthropod taxa recorded for the Azores (2,420; Borges et al. (2022b)). Put differently, a one-plot inventory captured nearly one-fifth of Pico’s known arthropod diversity and almost one-fourteenth of the Archipelago’s, which is a striking concentration of diversity at such fine spatial scale. This magnitude is consistent with island patterns showing that human-modified lowlands can host rich and compositionally distinct, arthropod assemblages (often with many exotics), while still retaining a substantial component of indigenous diversity and functioning as corridors or refugia within mosaic landscapes. Comparative work across Azorean habitats has repeatedly found: (i) higher indigenous richness in native forests, but substantial spillover into exotic forests and semi-natural pastures and (ii) strong compositional turnover from native forest to intensive pasture (e.g. Cardoso et al. (2009), Meijer et al. (2010), Picanço et al. (2017), Marcelino et al. (2021)). These patterns help explain why a small agroforest plot can accumulate such a large share of the island fauna while differing in composition from native-forest benchmarks.

On islands, well-designed sustainable agroforestry mosaics that embed native species and structural complexity can simultaneously sustain rich native/endemic assemblages, support pollinators and natural enemies, enhance connectivity amongst fragmented native remnants and provide early invasive-species surveillance, thereby reconciling production with conservation as a robust nature-based solution (NbS).

## Figures and Tables

**Table 1.** New arthropod records for Pico Island, Azores Archipelago. Entries list species (with authority and year), higher taxonomy, biogeographic Status, typical Habitat/Host, Likely pathway of introduction or spread, a brief Impact note and Evidence (voucher code and repository). Records are based on field detections cross-checked against the Azorean checklist (Borges et al. 2022b). The star (★) marks an archipelago-level first record. Status categories follow invasion biology usage: N - native non-endemic (native to the Azores, but also elsewhere), I - exotic/introduced (human-mediated arrival) and INDET -indeterminate/cryptogenic (uncertain origin). Pathway terms follow common usage in island biosecurity: plant trade/nursery stock, fruit/produce movement, commodity/storage, trade/transport and incidental with plants/soil (INC - Incidental transport with goods/ornamentals; COM - Commodity/storage pathway; PLANT - Plant trade/nursery stock).

Species	Order	Family	Status	Habitat/Host	Likely pathway	Impact note
*Neoscona crucifera* (Lucas, 1838)	Araneae	Araneidae	I	Anthropogenic sites; large orb webs	INC	Generalist predator; low direct economic impact
*Sitophilus oryzae* (Linnaeus, 1763)	Coleoptera	Dryophthoridae	I	Stored cereals; storage facilities	COM	Severe post-harvest losses
*Drosophila suzukii* (Matsumura, 1931)	Diptera	Drosophilidae	I	Soft-skinned fruits (cherry, strawberry, grape)	PLANT	Attacks ripening fruit; high crop losses
*Rivellia syngenesiae* (Fabricius, 1781)	Diptera	Platystomatidae	I	Herbaceous vegetation; adults on flowers	PLANT	Larvae feed on legume root nodules; potential effects on native Fabaceae
*Eristalis arbustorum* (Linnaeus, 1758)	Diptera	Syrphidae	N	Fields, gardens; adults on flowers		Important pollinator in natural and agricultural systems
*Xylota segnis* (Linnaeus, 1758)	Diptera	Syrphidae	N	Woodlands; adults on flowers; larvae in decaying plant matter		Pollination; larval role in nutrient cycling (saproxylic)
*Ceratitis capitata* (Wiedemann, 1824)	Diptera	Tephritidae	I	Broad range of fruit crops	PLANT	Major agricultural pest; quarantine relevance
*Protopulvinaria pyriformis* (Cockerell, 1894)	Hemiptera	Coccidae	I	Ornamentals and crops; greenhouses/gardens	PLANT	Can reach pest status under protected cultivation
*Pulvinaria floccifera* (Westwood, 1870)	Hemiptera	Coccidae	I	Broad tropical/subtropical hosts	PLANT	Horticultural pest; vigour/aesthetic damage
*Saissetia coffeae* (Walker, 1852)	Hemiptera	Coccidae	I	Coffee and many other hosts	PLANT	Economic damage on coffee/ornamentals
*Empicoris rubromaculatus* (Blackburn, 1889)	Hemiptera	Reduviidae	I	Various; predator of small arthropods	INC	Predatory; low risk to crops
*Ectomocoris chiragra* (Fabricius, 1803)	Hemiptera	Reduviidae	N	Generalist predator in varied habitats		Contributes to natural biological control
*Crossocerus elongatulus* (Vander Linden, 1829)	Hymenoptera	Crabronidae	INDET	Nests in earthy/crevice substrates; preys on Diptera; sometimes on flowers	INC	Predatory wasp; scant records in Azores
*Tetramorium caldarium* (Roger, 1857)	Hymenoptera	Formicidae	I	Disturbed, synanthropic sites; often indoors/greenhouses	INC	Nuisance indoors; greenhouse associate
*Polistes dominula* (Christ, 1791) ★	Hymenoptera	Vespidae	I	Synanthropic settings; varied habitats; social nests	INC	Potential impacts on native invertebrates, pollination webs, fruit production and public health
*Trigonidium cicindeloides* (Rambur, 1838)	Orthoptera	Trigonidiidae	I	Warm, humid microhabitats; often synanthropic	PLANT	Likely low risk
*Frankliniella occidentalis* (Pergande, 1895)	Thysanoptera	Thripidae	I	Ornamentals/horticultural crops; greenhouses	PLANT	Direct feeding damage; virus vector
